# Functional Genomics: New Insights into the 'Function' of Low Levels of Gene Expression in Stem Cells

**DOI:** 10.2174/138920210791616680

**Published:** 2010-08

**Authors:** Jennifer A Hipp, Jason D Hipp, Anthony Atala, Shay Soker

**Affiliations:** Wake Forest Institute for Regenerative Medicine, Wake Forest University School of Medicine, Medical Center Boulevard, Winston-Salem, NC 27157, USA

**Keywords:** Microarrays, differentiation, regenerative medicine, tissue engineering.

## Abstract

Understanding the global gene expression profile of stem cells and their multilineage differentiation will be essential for their ultimate therapeutic application. Efforts to characterize stem cells have relied on analyzing the genome-wide expression profiles that are biased towards the identification of genes that display the most pronounced differential expression. Rather than being viewed as a “blank” state, recent studies suggest that stem cells express low levels of multiple lineage specific genes prior to differentiation, a phenomenon known as “lineage priming.” It is not likely that low levels of lineage-specific genes produce sufficient amounts of differentiation factors, but rather to provide rapid transcription to a wide range of lineage programs prior to differentiation. Thus, stem cell differentiation may involve the elimination of other potential pathways and the activation of a specific lineage program.

## INTRODUCTION

The goal of regenerative medicine is to replace or restore normal function of cells, tissues, and organs that are damaged by disease. While progenitor cells are recognized as the ideal transplantation resource, cells obtained from diseased organs (congenital, cancerous or age-related effects) may not be appropriate for tissue engineering purposes. Furthermore, some primary cells cannot be expanded from particular organs, such as the pancreas and brain. In these situations, stem cells are envisioned as an alternative source of cells from which the desired tissue can be produced. Stem cells represent a source of versatile cells with the potential to replace diseased tissues and organs. 

According to data from the Centers for Disease Control, as many as 1 million Americans will die every year from disease that, in the future, may be treatable with cells derived from stem cells [1]. Diseases that might benefit from embryonic stem cell-based therapies included diabetes, heart disease, cerebrovascular disease, liver and renal failure, spinal cord injuries and Parkinson’s disease. The types of stem cells fall into three categories: embryonic, fetal and adult stem cells. Embryonic stem cells (ES cells) have the ability to grow indefinitely and differentiate into cells of all three germ layers. On the other hand, fetal stem cells are easily accessible and do not require technical manipulations, but they may not be as nimble as ES cells. Adult stem cells have a limited growth and differentiation potential. However, this is advantageous because they have a lower tendency to form tumors and mixed phenotypes. 

Despite the knowledge gained by stem cells’ ability to differentiate into multiple lineages, very little is known about the genes that govern the special properties of stem cells. Concerns about the clinical potential of stem cells include the quality of stem cell derivatives, the specificity of differentiation, and their tendency to form heterogeneous cell types. Understanding the molecular signature of stem cells will enable the control and direction of differentiation into particular phenotypes. Analysis of stem cell differentiation requires the expression of transcription factors and lineage-specific genes. However, it is becoming more important to test for negative markers of differentiation (whether stem cell-specific genes or other lineage-specific genes). Because of the recent advances of microarray technology, a non-biased view of the transcriptional status of stem cells and their derivatives can be determined. 

Microarray analysis measures the global expression of genes and can provide insight into the genetic programs expressed in stem cells. Microarrays can also be used to explore changes in gene expression during stem cell differentiation. Profiling stem cell differentiation in a lineage-specific and temporal-dependent manner may enable one to dissect the genetic wiring of differentiation. This review summarizes the methods used to study the expression profiles of stem cells and their differentiated derivatives and discusses possible mechanisms of stem cell differentiation. 

## METHODS TO CHARACTERIZE GENE EXPRESSION PROFILES OF STEM CELLS

With the ability to monitor the expression levels of almost every known and unknown gene, Affymetrix GeneChip technology is one of the most popular platform to study gene expression profiling [2, 3]. GeneChips are miniature platforms with approximately one million 25 base nucleotide sequences that enable measurement of the expression levels of 47,000 transcripts and variants including 38,500 well characterized human genes (HG-U133 Plus 2.0, www.affymetrix.com). The comercial platforms and their standardize protocols together with the ability to download of raw data allows one to reanalyze data generated in other studies for further exploration. For example, Stembase is a database of microarrays on stem cells and their derivatives to search novel stem cell markers and to search for genes that have unique functions in stem cells [4].

A simple microarray experiment looks for changes in gene expression at distinct timepoints of differentiation. Studies that characterize the progression of stem cells into specific lineages can be divided into two types of analysis; direct and indirect comparisons. The direct comparison, for example, compares sample A to sample B, and identifies genes that are differentially expressed between two samples [5]. This approach is biased towards the identification of genes that display the most pronounce differential expression between two samples however, genes that are expressed but have subtle changes in gene expression are ignored. On the other hand, microarrays can be use to define the molecular phenotype of a cell. Conventional analysis uses MAS5 (Microarray Suite version 5.0) algorithm for generating lists of genes that are present or absent but has been criticized for high false positive rates from exaggerated variances of genes with low levels of expression [6]. The reference design, or indirect comparison, is used because gene expression values can be meaningful in a relative sense [7]. Comparing stem cells to a reference file identifies genes that are over-expressed in stem cells with reference to a cell type. Genes that are over-expressed in stem cells could be considered as a genetic signature of stem cells. An alternative to MAS is Robust Multi-chip Average (RMA). Unlike MAS5, RMA identifies differentially expressed genes by comparing two cell types and reduces the variances of low intensity genes. 

The reference design approach has been widely used to characterize the changes in gene expression during embryonic stem cell differentiation. However, the type of reference materials varies from mixed RNA to RNA from a homogeneous cell type. The reference design was used to compare ESC profiles to a mixture of RNA obtained from various adult cells (universal RNA) [8]. This reference point is supposed to represent a signature of differentiated adult cells. Since microarrays assess the total RNA within a sample, microarrays containing a heterogeneous samples from mixtures may influenced by the predominating cell type. Another approach is to compared the ES profile to a single differentiated cell type, keratinocytes, to identify genes that are unique for ESC [8, 9]. 

## INSIGHTS INTO THE MECHANISM OF STEM CELL DIFFERENTIATION

Stem cells are an ideal resource for regenerative medicine. Despite the knowledge gained by stem cells’ ability to differentiate into multiple lineages, very little is known about the genes that govern the special properties of stem cells. Stem cells have been previously thought of as a “blank” cell and the activation of lineage-specific programs indicates commitment into a particular lineage. However, it has been shown that embryonic stem cells express more genes than their differentiated derivatives [9]. In addition, studies have shown that various types of stem cells express low levels of genes associated with multiple lineages prior to differentiation, a phenomenon known as “lineage priming” [10]. It is unclear whether the expression is due to non-specific hypertranscription or to allow a rapid up-regulation of a single lineage program when cells differentiate into a particular lineage [11]. 

## EMBRYONIC STEM CELLS EXPRESS TISSUE-SPECIFIC GENES

Embryonic stem cells are a source of pluripotent stem cells that can be used for regenerative medicine. Unlike adult stem cells, embryonic stem cells have been shown to differentiate into at least 200 somatic cell types, such as dopaminergic neurons and beta islet cells [12, 13]. Embryonic stem cells (ESCs) are derived from the inner cell mass at the blastocyst stage of a fertilized embryo. ESCs have the ability to differentiate into all somatic cell types of an organism. In addition, ESCs possess the ability to self-renewal indefinitely and provides an unlimited source of cells for cell therapy. Since the inner cell mass is obtained by immuno-isolation, the isolation of ESCs destroy the embryo. This has stimulated research on generating alternative sources of pluripotent stem cells. It has been recently shown that adult fibroblasts can be reprogrammed into pluripotent stem cells (iPS) by exogenous transcription factors. Although the properties of iPS cells have been compared to those of ESCs, the gold standard, a better understanding of ESCs is needed before they can be compared to iPS and their clinical potential. 

Initial efforts have been made to define “stemness,” a set of genes that are commonly expressed in multiple stem cell types, to provide insight underlying self-renewal and the ability to differentiate into multiple lineages [14]. However, ascribing stem cell functions from gene lists is analogous to taking a car apart and explaining how it works. This approach focuses on the genes themselves rather than deciphering potential mechanisms of stem cell differentiation. Rather than identifying genes that are common between multiple stem cell types, a better approach is to characterize individual stem cell types in order to gain a better understanding of a stem cell identity. 

Microarrays were used to identify a molecular signature of ESCs by profiling 6 human ESC lines (Bhattacharya 2004). Using arrays that were fabricated in-house by spotting oligonucleotides, gene expression patterns of ESC lines were compared to expression patterns of human universal RNA. Hierarchical clustering analysis, which is an unsupervised method to find biologically significant patterns of gene expression, showed that 6 ESC lines clustered tightly together, indicating a similar expression profile and express several genes known to be expressed in human ES cells including OCT4, NANOG, TDGF1, GALALIN, CONNEXIN 43. Some known markers of undifferentiated ES cells did not meet the cutoff criterion (expression in all 6 lines). These genes included, CD24, DNMT3B, SOX2 were expressed in 4 cell lines. Interesting, 5 genes thought to be specific for differentiation were present at high levels in all 6 ESC lines (keratin 8, keratin 18, beta tubulin 5, cardiac actin, and troponin T1) and were confirmed by RT-PCR. Interestingly, some researchers have noted differences in the behavior of cell lines [15]. Since this type of analysis identifies genes that are enriched in all 6 embryonic stem cell, maybe the differences in their behavior could be related to the unique expression of genes of each line. The unique expression of genes in each line could also be due to the fact that ESCs spontaneously differentiate and that tissue-specific gene expression could be due to the contamination of differentiated ESCs. 

Another study used microarrays to study the genetic programs expressed in human ESCs by comparing transcriptional profiles of ESC to progenitor and mature cells of the hemapoietic and ketatinocytic lineages to provide snapshots of differentiation [9]. Using affymetrix arrays and MAS5, 4,450 probesets were detected as significantly expressed in ESCs while 3,000 probesets were expressed in the differentiated state. This suggests that ESCs express more genes than adult cells. Tissue classification of the 4,450 probesets identified in ESCs showed that 700 probesets are not expressed in adult tissues, 3,300 probesets are expressed in multiple adult tissues, and around 1000 probe sets are tissue-specific genes. 

Elevated transcriptional activity has been observed in embryonic stem cells (Efroni S, 2008). Here, transcriptional activity, measured by [H3] uridine incorporation in ESCs and 7 day neural progenitor cells (NPCs) derived from ESCs, were almost 2-fold higher in ESCs compared to NPCs. In addition, the transcriptional status of several tissue-specific genes was assessed in ESCs. Transcripts for 11 out of 12 lineage-restricted genes expressed in ESCs at low levels and include actin alpha 1, receptor activator of NF kappa B ligand, prostate androgen induced 1, small proline rich protein 2A, albumin, CD3, CD8, glial fibrillary acidic protein, surfactant protein B, uromodulin, synaptotagmin 1, myogenin). In addition, they compared the transcription levels of tissue-specific genes at different stages of neuronal differentiation and found that levels of transcription decreased during differentiation. As ESCs progressed into NPCs and postmitotic neurons, 8/12 and 5/12 of the transcripts were expressed respectively. Protein was not detected by western blot analysis. The data suggests that as stem cells differentiate into more mature cells, the expression of multiple tissue-specific genes decreases.

### Hematopoietic Stem Cells

Promiscuous genes have also been observed in the hematopoietic system, where expression of genes of multiple lineages was detected prior to commitment [16]. The hematopoietic system is the best studied stem cell system, demonstrating the stage-specific steps of differentiation of HSCs into multiple lineages and their ability to repopulate the entire hematopoietic system from a single cell. Molecular analysis at each step of differentiation has identified several crucial regulators, transcription factors and genes as HSC differentiate into each hematopoietic lineage. It is unclear how undifferentiated HSC can maintain their ability to differentiate into mutliple lineages. To test the hypothesis that HSC are primed to express multiple lineage-affiliated programs, Hu *et al. *showed that HSC express erythroid (globin) and myeloid (myeloperoxidase) gene expression programs by single cell RT-PCR. Further analysis of globin and myeloperoxidase gene expression was assessed in more differentiated cell types. Globin was expressed in erythroid-committed cells but not myeloid-committed cells, likewise, myeloperoxidase was expressed in myeloid-committed cells but not erythroid-committed cells. This data suggests that as cells commit to a specific lineage, their promiscuity for multiple lineages disappears. This supports the idea that primitive stem cells express a more diverse set of lineage programs and the number of lineages decrease as cell become more committed into a specific lineage. 

Since single cell analysis are limited to analysis of selective set of genes, a global view of gene expression profiles can provide insight into the molecular components involved in differentiation. However, few studies have performed microarray analysis on single cells after RNA amplification. But one of the limitation RNA amplification is its 3’ bias, laborious, and has the potential to distort relative transcript abundances [17]. Thus, microarray analysis of purified clonal populations of HSCs can provide insights into the global gene expression profiles of HSCs. Akashi *et al. *profiled purified HSCs, non-self-renewing multipotential progenitors (MPPs) and lineage-restricted (common lymphoid progenitors) CLPs and common myeloid progenitors (CMPs) [18]. HSCs expressed genes specific to nonhematopoietic tissues including brain, liver, heart, kidney, muscle, and endothelium. The number of genes specific to nonhematopoietic tissues decreased in MPPs, CMPs and CLPs.

Are stem cells that are primed really stem cells or partially differentiated? Ye *et al*. generated a cre-lox approach to follow the fate of HSCs that expressed a lineage-affiliated marker [19]. Mice were generated with a yellow fluorescent protein in the lysozyme gene, which is highly expressed in myeolomonocytic cells. Using *in vivo* lineage tracing technique, it was shown that lysozyme is expressed at low levels in a subset of HSCs and are capable of long-term repopulation potential. This suggests that the expression of a myeloid gene does not abolish their stem cell potential. 

### Transdifferentiation

Transdifferentiation is a process where a cell is committed towards one lineage switches into a cell type of a different lineage. The ability for a mature cell to change phenotypes has remained controversial because of cell fusion and progenitor cell contamination. Using mesenchymal stem cells as a model to study transdifferentiation, Song *et al. *showed that fully differentiated osteoblast derived from MSCs were capable of differentiating into adipocytes and chondrocytes [20]. To ensure that the osteogenic differentiation of MSCs did not result in mixed phenotypes and were not contaminated with progenitor cells, MSCs were transfected with GFP driven by the osteocalcin promoter and sorted by fluorescence activated cell sorting (FASC). Although only 5% of the cells expressed GFP, all cells expressed alkaline phosphatase activity. When cells were induced into a chondrogenic lineage, 97% of the cells stained positive for collagen II and proteoglycan link proteins. This data suggest that fully differentiated cells can switch phenotype in response to environmental signals. 

Other types of stem cells have also exhibited lineage priming. Muscle-derived stem cells (MDSCs) express myogenic and stem cell markers but are negative for hematopoeitic markers (c-kit, CD45) and other blood-lineage markers (Mac-1, Gr-1, CD3, CD4, CD5, CD8) [21]. MDSCs can differentiate into myotubes and hematopoietic cell types [22] but its not clear whether MDSCs preserve their myogenic potential after differentiation into hematopoietic lineages. It was determined that MDSCs were able to repopulate the bone marrow of lethally irradiated mice. Donor MDSCs-derived hematopoietic cells that were isolated from bone marrow of the recipient animals and purified by the expression of the neomycin resistance gene were still able to form myotubes *in vitro* and *in vivo*. Thus, these MDSCs were able to preserve their myogenic potential after transplantation when isolated from the bone marrow of primary recipient mice and retransplanted into the skeletal muscle of a secondary recipient mice. 

## CONCLUSIONS

While low expression of lineage-specific genes may not inhibit differentiation into other lineages, it is difficult to determine the expression levels that are necessary to induce stem cells into a particular pathway for a specific lineage. The over expression of MYC, OCT4, SOX2 and KLF4 has been shown to reprogram fully differentiated cells into pluripotent stem cells, and up to 20 viral integrations are present in each clone [23]. On the other side of the spectrum, recombinant HoxB4 fusion proteins can be used to push embryonic stem cells towards a hematopoietic lineage [24]. The overexpression of genes can also change phenotypes but again, it is not clear what level of expression is necessary. A recent study showed that the dose-dependent effect of OCT4 expression has dual effects; basal levels of OCT4 while the up-regulation of Oct4 induces a cardiogenic fate by turning on Sox17, Hex, Wnt3a and BMP2 [25]. This suggests that the dosage of Oct4 is critical for maintaining pluripotency or driving cells into a more differentiated state. 

Microarrays are a common technique to characterize the molecular signatures of stem cells. A newer and more comprehensive approach to analyze transcription is RNA sequencing. Sequencing the RNA content of cells can provide information to quantify gene expression and identify single-nucleotide polymorphism (SNP), novel transcripts, novel isoforms, and rare transcripts in a single experiment [26,27]. Sequence data is mapped to a reference genome and counted, thus the total number of “reads” for a given transcript is proportional to the expression level of RNA. Since quantification is based on direct sequencing rather than hybridization of fluorescent probes, low abundant transcript can be reliably identified due to the low background noise of RNA sequencing [28]. Sequencing-based approaches have the advantage of identifying both known and unknown genes in contrast to microarrays, where oliogonucloetides must be present on the platform. However, limitations of RNA sequencing include the high cost and long turnaround time. Yet, the comprehensive genomic information obtained from RNA sequencing is enormous and may outweigh these deficiencies. 

Before stem cells can be used as any type of clinical therapy, strict guidelines must be established to ensure the quality of the cells, the specificity of differentiation, and the assessment of mixed phenotypes. While lineage-specific gene expression and cell surface markers are commonly used to describe a differentiated phenotype, global gene expression profiling is necessary to provide a non-biased evaluation of the quality of cells. Genome-wide characterization of stem cells suggests that stem cell express genes that represent multiple lineage-specific programs. Thus, the molecular basis of stem cells appears to entail a promiscuous gene expression pattern and the expression of these multiple lineage-specific programs may reflect the potential of stem cells to development into these lineages. The therapeutic potential of stem cells largely relies on understanding the molecular signature of stem cells and their derivatives. 
